# Live Poultry Trading Drives China's H7N9 Viral Evolution and Geographical Network Propagation

**DOI:** 10.3389/fpubh.2018.00210

**Published:** 2018-07-27

**Authors:** Ruiyun Li, Tao Zhang, Yuqi Bai, Haochuan Li, Yong Wang, Yuhai Bi, Jianyu Chang, Bing Xu

**Affiliations:** ^1^State Key Laboratory of Remote Sensing Science, College of Global Change and Earth System Science Beijing Normal University, Beijing, China; ^2^Ministry of Education Key Laboratory for Earth System Modeling, Department of Earth System Science Tsinghua University, Beijing, China; ^3^State Information Center Beijing, China; ^4^Chinese Academy of Surveying and Mapping Beijing, China; ^5^CAS Key Laboratory of Pathogenic Microbiology and Immunology, Institute of Microbiology Chinese Academy of Sciences, Beijing, China; ^6^College of Veterinary Medicine China Agricultural University, Beijing, China

**Keywords:** H7N9, live poultry trade, network, evolution, propagation

## Abstract

The on-going reassortment, human-adapted mutations, and spillover events of novel A(H7N9) avian influenza viruses pose a significant challenge to public health in China and globally. However, our understanding of the factors that disseminate the viruses and drive their geographic distributions is limited. We applied phylogenic analysis to examine the inter-subtype interactions between H7N9 viruses and the closest H9N2 lineages in China during 2010–2014. We reconstructed and compared the inter-provincial live poultry trading and viral propagation network via phylogeographic approach and network similarity technique. The substitution rates of the isolated viruses in live poultry markets and the characteristics of localized viral evolution were also evaluated. We discovered that viral propagation was geographically-structured and followed the live poultry trading network in China, with distinct north-to-east paths of spread and circular transmission between eastern and southern regions. The epicenter of H7N9 has moved from the Shanghai–Zhejiang region to Guangdong Province was also identified. Besides, higher substitution rate was observed among isolates sampled from live poultry markets, especially for those H7N9 viruses. Live poultry trading in China may have driven the network-structured expansion of the novel H7N9 viruses. From this perspective, long-distance geographic expansion of H7N9 were dominated by live poultry movements, while at local scales, diffusion was facilitated by live poultry markets with highly-evolved viruses.

## Introduction

The detection of a novel H7N9 virus in March 2013 was the first time that a low-pathogenic avian influenza A(H7N9) virus was identified in humans (1). Since then, the disease has continued to evolve in avian and humans, causing 766 confirmed human infections in 19 provinces of mainland China in the following 3 years.^1^

Recent analyses demonstrated that the novel H7N9 virus was a reassortant, with surface and internal gene segments originating from wild birds and the H9N2 lineage in poultry, respectively. This indicates that wild birds were the most likely source of infection, introducing the virus into domestic ducks and chickens through sequential reassortment events, with a consequent spillover to humans by means of live poultry exposure (2–9). The resulting multiple H7N9 lineages and genotypes suggest that the evolution of H9N2 has facilitated the genesis of the internal segments of this novel reassortant (4, 10, 11). It has therefore exhibited greater genetic diversity compared with the surface genes (4, 12). As our preliminary researches have pointed out, this interaction at the wild birds–poultry–humans interface was common in the spread of infectious diseases on various scales (13–17).

**Figure 1:**
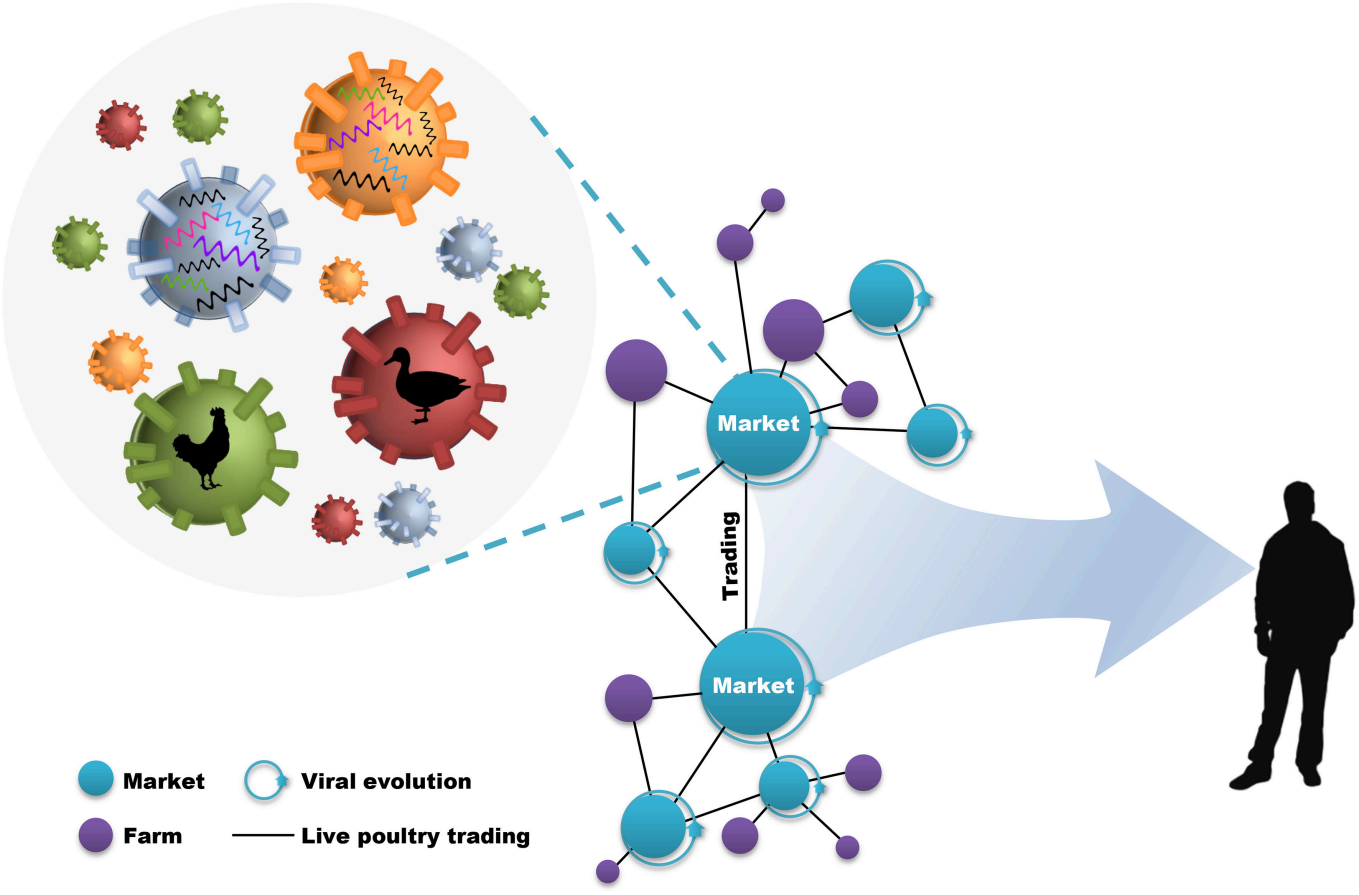
Live poultry trading network drives viral evolution and poultry–humans sequential transmissions. The live poultry trading network consist of live poultry markets, farms, and the trading among them. The genetic evolution of avian influenza viruses is facilitated by the co-circulation of different viral subtypes and hosts within markets, and further expanded by poultry trading. This poultry trading framework increases the potential for poultry–humans sequential transmissions.

During this process, live poultry trading network enclosing live poultry markets (LPMs) and live poultry transportations (LPTs), was vitally important to the further genetic evolution of avian influenza viruses (AIVs) and the poultry–humans sequential transmissions (Figure 1). LPMs were ideal environment for the co-circulation and co-infection of different subtypes and the spillover to humans (18–20). However, it will be efficient in the reduction of daily number and growth rate of new human cases through target control measures (e.g., market closure) (21, 22). Nevertheless, interventions mainly focused on LPMs was insufficient in restricting the geographic expansion of the viruses. The fact that the transmission of H7N9 virus into regionally-specific H9N2 gene pools and the consequent establishment of more diversified genotypes can be accelerated by the inter-provincial trading of live poultry (4, 8). In this case, preventions of viral spread along poultry movements should also be implemented practically. However, although restrictions in LPMs and LPTs have been put forward as one of the important prerequisites in limiting viral expansion, it was unclear about how live poultry trading shapes the geographic propagation of viruses.

Previous studies have reported that interdisciplinary datasets and approaches provide a unique opportunity to infer the dynamic footprints and underlying mechanisms of AIVs (14, 17). In the present study, we employed sequence data and social-economic information to demonstrate the position of live poultry trading in dominating the local evolution and spatial diffusion structure of H7N9 viruses.

## Materials and methods

### Ethics approval statement

All animal work was approved by the Beijing Association for Science and Technology (approval SYXK [Beijing] 2007-0023). The laboratory animal research was performed in the microbiology laboratory of China Agricultural University, and in accordance with Beijing Laboratory Animal Welfare and Ethics guidelines issued by the Beijing Administration Committee of Laboratory Animals and China Agricultural University Institutional Animal Care and Use Committee guidelines (ID: SKLAB-B-2010-003).

### Sequence data and analysis

Samples were collected from two different LPMs in Jiangxi Province during June 2014. These viruses were isolated from positive samples and sequenced following the high-throughput sequencing on an Illumina Hiseq2500 sequencer. The virus isolation rate was defined as the number of H9N2 samples divided by the total number of samples we collected.

Sequence data for all the internal gene segments of H7N9 isolates in March 2013–December 2014 and those of H9N2 isolates in January 2010–December 2014 were obtained from the GenBank database of the National Center for Biotechnology Information (23). The H9N2 isolates reported in previous studies were also included for the categorization and identification of the closest lineage to H7N9 isolates (11, 24). The integrated dataset including all these published H7N9 and H9N2 sequences and those we collected was used for further analysis. To address the issue of the unbalanced number of sequences among isolation locations, we reclassified locations by combining the geographically adjacent and social-economically similar provinces into an integral region. This location reassignment generated six regions/provinces for H9N2 isolates: Beijing-Tianjin-Hebei region (J-J-J region), Shanghai-Zhejiang region, and Shandong, Jiangsu, Jiangxi, and Guangdong Province. Since no H7N9 isolates were collected from J-J-J region and Shandong Province, the location of H7N9 isolates were assigned to one of the other four regions/provinces. In this study, both H7N9 and H9N2 isolates were assigned a specific information, containing the viral subtype (i.e., H9N2 or H7N9), spatial location (i.e., region or province) and sampling location (i.e., LPMs, farms, or others), and the additional epidemic waves for H7N9 isolates. In addition, isolates sampled from LPMs and farms were extracted to quantify and compare the substitution rates, or the rate at which mutations fix in a population, between subtypes and sampling locations.

The phylogenetic analysis of these sequence data was implemented to identify the evolutionary structure using Molecular Evolutionary Genetics Analysis (MEGA) software version 6.0 (25). In order to ensure the identity of phylogenetic topology, phylogenetic tree was constructed by both the maximum likelihood and neighbor-joining distance-based matrix algorithms. The H9N2 isolates during 2010–2014 was then grouped into each lineage, and those shared the highest similarity with H7N9 viruses were defined as the closest H9N2 lineage to H7N9 viruses (Supplementary Table 1).

The inference of the discrete phylogeography of internal segments of H7N9 and closest H9N2 was made in the Bayesian statistical inference framework using a Bayesian Markov chain Monte Carlo (MCMC) method in the BEAST package (version 1.8.0) (26, 27). In addition, substitution rate of isolates sampled from LPMs and farms were also estimated using strict molecular clock, Bayesian skyline coalescent prior, and HKY85+Gamma nucleotide substitution model. The convergence diagnosis of MCMC chain was inspected in Tracer (http://beast.community/tracer) by the trace plot of 20,000,000 iterations, with the effective sample size (ESS) greater than 200. The uncertainty analysis of alternative molecular evolutionary processes was also implemented by comparing the performance of different evolutionary models (i.e., the combination of molecular clock models and coalescent priors) using Bayes factor (Supplementary Table 2).

### Risk factors and market accessibility

The set of risk factors in this study included national road density, the density of live poultry markets, and the relative production and consumption of poultry for the above regions/provinces. The national road data were extracted from the 1:1,000,000 geomatics dataset provided by the China State Information Center. The length of the pairwise national road between two locations refers to the total length of all the alternative national roads between them. In addition, on the basis of the big data for public health, the information of LPMs was derived from the search engine of Chinese Academy of Surveying and Mapping. This automated data mining process was executed with different terms, i.e., live poultry, agricultural market, fresh market, live bird market (Supplementary Table 3), and the replicated records were then removed. The collected pairwise road distance and number of LPMs were further transformed into national road density and LPMs density (i.e., the length of national road and the number of LPMs per square kilometers, respectively). The relative production and consumption of poultry in 2010–2013 were collected from the China Statistical Yearbook (28).

The market accessibility was defined as national road density to represent the transportation cost between pairwise markets. Specifically, the higher density of national road indicates the multiple alternative transportation approaches, and hence more ease and higher feasibility for live poultry trading.

### Geographic structure of H7N9 genetic differentiation

The Fixation index (*F*_*ST*_) was derived from the *F*-statistics proposed by Sewall Wright to study population structure (29). Here, we used *F*_*ST*_ to quantify the amount of genetic variance that can be explained by market accessibility. The value of *F*_*ST*_ varied from 0 and 1, and the two extreme estimates 0 and 1 indicated no and complete spatial structure of genetic variations, respectively. Specifically, a modified *F*_*ST*_ defined as $F_{ST}=1-\frac{H_{w}}{H_{b}}$ was used (30), where *H*_*w*_ and *H*_*b*_ are the average genetic variations within and between provinces *i* and *j*, defined as

(1) $$\begin{array}{rcl} H_{w} & = & \frac{\left(\frac{2}{n_{i}\left(n_{i}-1\right)}\underset{ix<iy}{\sum }\delta_{ix,iy}\right)+\left(\frac{2}{n_{j}\left(n_{j}-1\right)}\underset{jx<jy}{\sum }\delta_{jx,jy}\right)}{2} \end{array}$$

(2) $$\begin{array}{rcl} H_{b} & = & \frac{\underset{ix<jy}{\sum }\delta_{ix,jy}}{n_{i}n_{j}} \end{array}$$

where δ_*xy*_ is the pairwise genetic distance between two nucleotide sequences (*x* and *y*); *n*_*i*_ and *n*_*j*_ are the numbers of sequences in *i* and *j*, respectively; and *ix* and *jy* are the *xth* and *yth* sequences in province *i* and *j*.

For each pair of locations with H7N9 isolates, we first calculated its *F*_*ST*_ and then proceeded to implement the linear regression and make an initial investigation of the relations between market accessibility and genetic divergence. Mantel test (31) was further employed to evaluate the strength of this genetic-geographic relations (Supplementary Figure 1).

### Poultry transportation and viral migration network

Poultry transportation information was gathered from news reports on websites and statistical reports. The subtle intra-provincial movement routes were then combined or eliminated to give an illustrative inter-provincial transportation network. The corresponding undirected graph with only paths between regions/provinces was also generated for the subsequent correlation test with viral migration routes.

“Markov jump” counts (32) which provided an estimate of the expected number of spatial location transitions based on the reconstructed phylogeny, established the gene flow network among locations. With this quantitative assessment, we depicted a H9N2–H7N9 viral migration network for each internal gene segment to reflect the cross-subtype interactions and the H7N9 spatial pattern of spread. Statistically supported transmission paths were selected based on Bayes factor, with cutoff values of 3. These proposed networks were further merged into a single viral dynamic network where linkage type and strength indicated the persistence and the number of occurrence in all internal segments, respectively. This integrated network was then abstracted into an undirected viral propagation graph with only inter-provincial routes, to test the similarity with the structure of poultry transportations.

Considering the alternative viral migration process along unobserved networks, 1,000 random graphs were generated to ascertain the statistical significance of correlations between the topology of two graphs via quadratic assignment procedure (QAP) test (33).

## Results

### Sampling at LPMs in Jiangxi province

Four hundred and ninety three samples were collected from two LPMs in Jiangxi Province during June 2014, among which 61 samples (12.37%) were H9N2-positive. Besides, the mixture sample of H9N2 and other subtypes were also identified, with the isolation rate to be 7.52% for the mixture of H9N2 and another subtype and 2.84% for those mixed with two or more subtypes.

### Substitution rate of viruses in LPMs and farms

Generally, both H7N9 and H9N2 viruses sampled during March 2013–December 2014 substituted at a higher rate compared with those in previous years (i.e., January 2010–December 2012), especially for viruses obtained in LPMs (Table 1). It is also noted that all the internal segments of H7N9 evolved even faster than the contemporary H9N2 viruses, particularly for the NS gene.

**Table 1:** Mean substitution rates^a^ of avian influenza samples isolated in different time periods and locations.

**Segment**	**H7N9**	**H9N2 (March 2013-Dec. 2014)**			**H9N2 (Jan. 2010-Dec. 2012)**		
	**LBMs**	**Farms**	**LBMs**	**Total^b^**	**Farms**	**LBMs**	**Total**
PB2	6.15	5.87	4.30	5.75	2.48	3.75	3.10
PB1	5.81	4.06	2.78	4.09	3.34	3.97	3.74
PA	5.36	5.04	3.75	5.13	4.52	2.49	2.16
NS	12.20	5.23	2.39	5.30	5.76	2.70	3.73
NP	7.06	4.45	2.45	4.29	3.15	3.51	2.86
MP	6.55	4.73	1.86	4.82	3.06	2.60	1.95

a *The rate at which mutations fix in a population (unit: × 10^-3^ substitutions/site/year)*.

b *Total: isolates sampled from both LBMs and farms*.

### H7N9 genetic differentiation structured by market accessibility

Statistically significant associations were observed between the genetic divergence and accessibility among markets, with the correlation coefficient values (*R*) of regression ranged from 0.54 to 0.76 (Supplementary Figure 1). There was a general decreasing trend of the regional genetic differentiation level with the increasing market accessibility. This result implied that market accessibility, or the feasibility of poultry trading, predominantly drove the genetic differentiation structure. Additionally, the higher road density benefited the live poultry trading by providing multiple alternative approaches and hence lowering the costs of transportations. However, the residual genetic variation not explained by the regression (around 24–46%) suggested that factors other than national road density may have also influenced the spatial differentiation pattern of internal genes.

### Spatial structure of H9N2–H7N9 viral propagation

**Figure 2:**
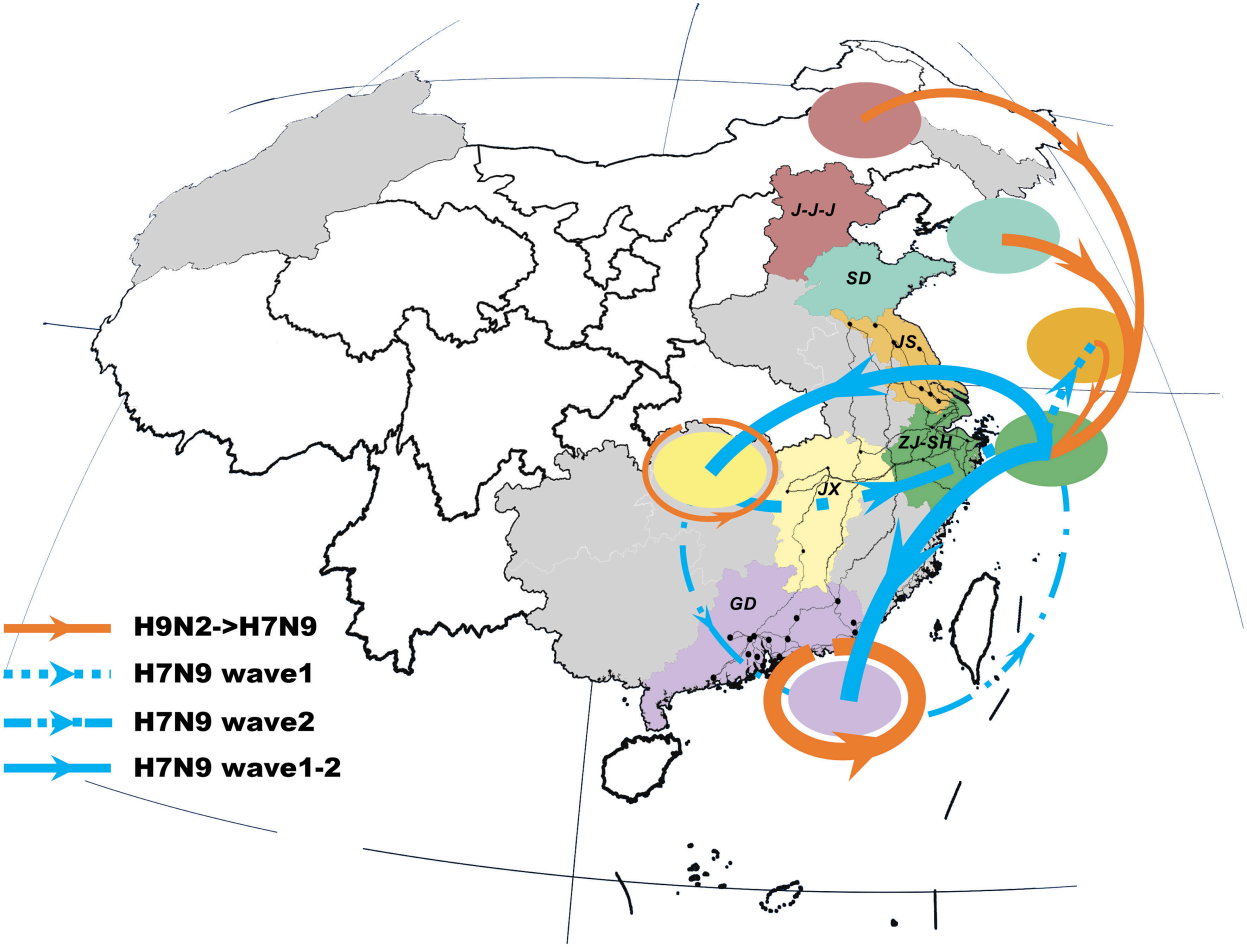
Viral propagation network for internal gene segments of H9N2-H7N9 viruses. Viral migration routes are distinguished by subtype, epidemic wave, and strength. Specifically, orange ones represent interaction between H9N2 and H7N9 viruses; while the blue dot lines, dash-dot lines and solid lines with arrow indicate the viral migration pattern of H7N9 in wave 1, wave 2 and persist during two epidemic waves, respectively. The linkage strength is represented by the number of occurrence in all internal segments. Locations with H7N9 human cases included in the study are colored and those not included are shaded gray. The primary national roads and cities with H7N9 human cases are represented by black lines and black dots, respectively. J-J-J, Beijing–Tianjin–Hebei region; SD, Shandong; JS, Jiangsu; SH-ZJ, Shanghai–Zhejiang region; JX, Jiangxi; GD, Guangdong.

The previous H9N2 viruses were closely related to the newly-emerged H7N9 viruses, forming a distinct north-to-east inter-provincial transmission route and a locally-evolved pattern in the southern region (Figure 2). Specifically, the H9N2 viruses that dispersed from northern area (i.e., J-J-J region and Shandong Province) may be the donor of the H7N9 internal genes in the Shanghai–Zhejiang region. However, we also noted that the H7N9 viruses in Jiangxi and Guangdong were more closely related to local than to external H9N2 viruses.

The spatial dispersion patterns of the H7N9 viruses were diverse and highly-structured, with characteristics specific to each epidemic wave (Figure 2 and Table 2). In particular, the spatial spread of the H7N9 viruses was predominantly restricted to the vicinity of the Shanghai–Zhejiang region during the early period of wave 1, with only a small fraction reaching Jiangsu (the dot line with arrow). However, the long-distance spread from Shanghai–Zhejiang region to Jiangxi and Guangdong Provinces occurred at the end of wave1 and persisted as the major routes until the next wave (the solid lines with arrow). This wide-range migration was followed by the apparent backward transmission from Jiangxi and Guangdong Provinces to Shanghai–Zhejiang region and the established linkage between Jiangxi and Guangdong Province (the dash-dot lines with arrow). Thus, the viruses in the east and south region was linked through the bidirectional or circular transmission between them.

**Table 2:** Statistical performance of viral propagation paths.

**Segment**	**H9N2-H7N9**			**H7N9**		
	**Transmission**	**Transitions**	**BF**	**Transmission**	**Transitions**	**BF**
PB2	J-J-J → SH-ZJ	3.58	51.62	SH-ZJ → JX	1.23	>100
	GD → GD	4.44	36.28	JX → SH-ZJ	2.94	33.18
	SD → SH-ZJ	5.45	35.42	SH-ZJ → GD	4.49	3.89
PB1	SD → SH-ZJ	6.90	>100	JX → SH-ZJ	2.43	59.87
	JX – JX	2.09	60.92	SH-ZJ → GD	2.12	18.97
	GD → GD	2.63	16.97	SH-ZJ → JX	2.56	17.66
PA	SD → SH-ZJ	8.53	>100	SH-ZJ → GD	1.04	>100
	GD – GD	3.23	39.37	SH-ZJ → JX	1.74	49.96
				SH-ZJ → JS	2.88	6.56
				GD → SH-ZJ	1.31	5.44
				JX → SH-ZJ	2.76	4.78
				JX → GD	2.81	69.77
NS	J-J-J → JS	1.09	50.55	JX → SH-ZJ	2.37	99.94
	JS → SH-ZJ	2.88	49.70	SH-ZJ → JX	3.09	60.64
	GD – GD	2.10	13.32	SH-ZJ → GD	2.47	12.04
				SH-ZJ → JS	1.48	3.09
NP	J-J-J → SH-ZJ	10.00	>100	SH-ZJ → JX	1.37	34.53
	SD → SH-ZJ	1.97	>100	SH-ZJ → JS	1.48	23.49
	GD – GD	2.37	33.14	SH-ZJ → GD	1.84	3.04
MP	JX → JX	2.17	70.25	SH-ZJ → GD	5.14	>100
	JS → SH-ZJ	2.44	50.74	GD → SH-ZJ	1.68	45.31
	GD – GD	2.11	45.41	JX → GD	1.36	39.95
	J-J-J → SH-ZJ	1.51	44.29	SH-ZJ → JX	1.13	4.71

*Statistically supported transmission paths were selected and sorted by Bayes Factor, with cutoff value to be 3. Transmissions, or the expected number of location state transitions along branches of the inferred phylogenies is measured by “Markov jumps” counts. JS, Jiangsu; SH, Shanghai; ZJ, Zhejiang; GD, Guangdong; JX, Jiangxi; SD, Shandong; J-J-J, Beijing-Tianjin-Hebei region; BF, Bayes Factor*.

### Correlation between viral transmission and LPTs

**Figure 3:**
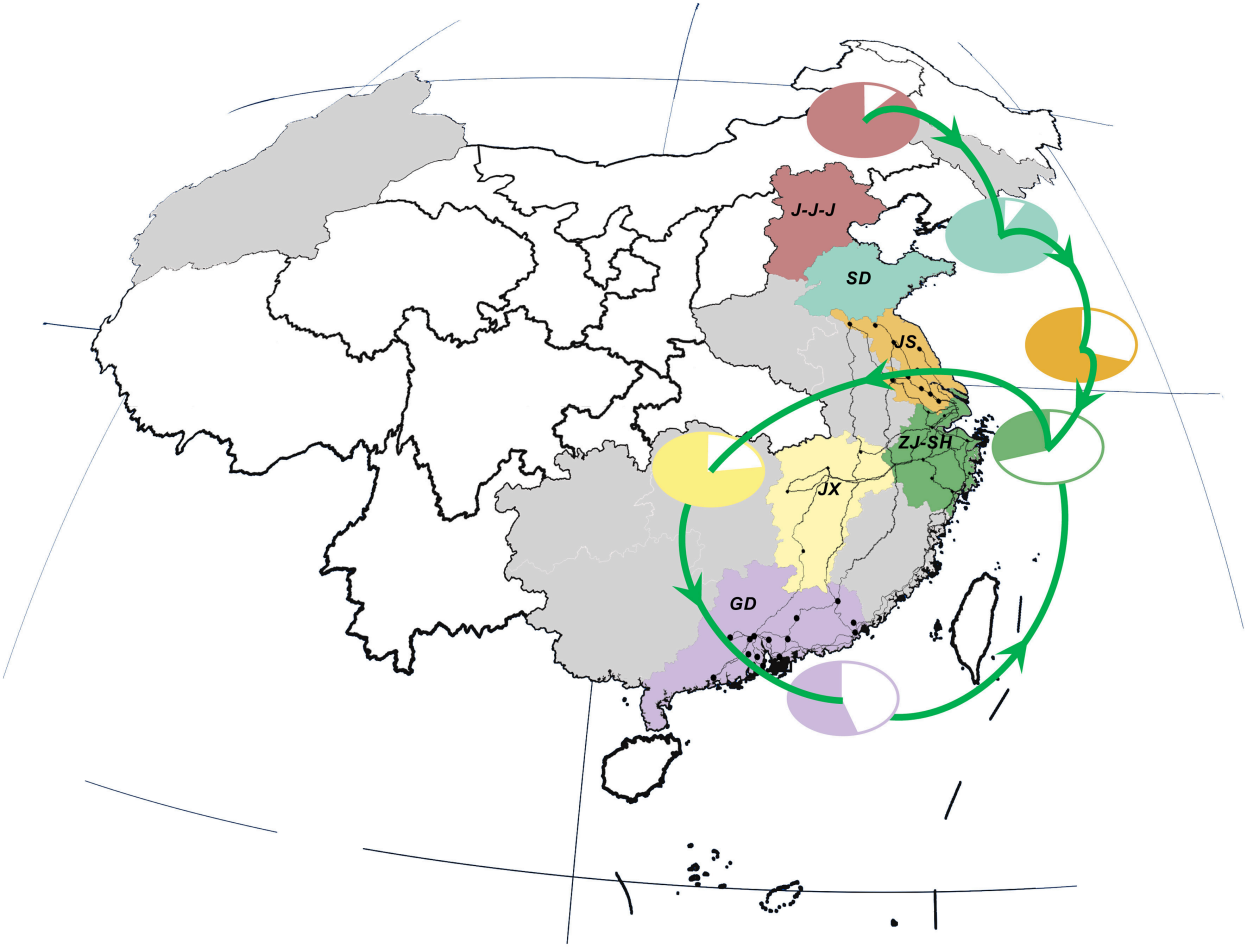
Live poultry transportation network and relative poultry production and consumption in study area. Locations, national roads and cities are colored in consistent with those in Figure 2. Poultry transmission paths are illustrated in green lines with arrows. The relative percentage of poultry production (color area) and consumption (white area) in each region/province is distinguished by the same color as the corresponding locations.

Interestingly, there was a strong similarity between the pattern of viral propagation and LPTs. More precisely, the poultry movement path, which originated from primary poultry production area in the north to the main poultry consumption region in the east (i.e., Shanghai–Zhejiang region) (Figure 3), was consistent with the north-to-east spread pattern of AIVs. Furthermore, the circular poultry transportation paths connecting the Zhejiang–Shanghai region with Jiangxi and Guangdong Provinces were also similar to and may therefore have forced the H7N9 dissemination routes among them.

This similarity was further verified by the result of correlation analysis which rejected the hypotheses of random viral migration process (0.764, *p*-value: 0.037) (Supplementary Figure 2), and hence indicated a statistically significant association between the topology of viral diffusion and poultry movement graphs. In other words, the observed pattern of gene flow was highly-structured and can be best fitted by the migration process along the LPTs network.

## Discussion

Our results demonstrated that the network-structured diffusion of the internal gene segments of AIVs followed and can therefore be explained by the consistent inter-provincial paths of live poultry trading. However, the mode or mechanism of poultry trading in shaping the inter-subtype viral interactions differed between locations. More specifically, the genesis of H7N9 in eastern China may have been facilitated by the previous H9N2 viruses disseminated from the northern region along poultry movement paths (11). Contrast with this inter-regional viral interaction, the localized H9N2–H7N9 transmission contributed to the evolution of H7N9 in Guangdong Province. Thus, the H7N9 virus endemic in Guangdong may jointly be resulted from the continuous reassortment with local environmental and avian H9N2 viruses (22, 34, 35) and the H7N9 gene flow through circular poultry movements. The time-specific spatial propagations of the H7N9 viruses suggested that each location played distinct yet crucial role during different epidemic stages. In the first epidemic wave, Shanghai–Zhejiang may have acted as the epicenter, primarily diffusing virus to the adjacent provinces and introducing the viruses into Jiangxi and Guangdong Provinces. This dominant position was then taken over by Guangdong, with a higher emigration rate (12) and backward south-to-east dynamics during the second wave. These two established epicenters of novel H7N9 paralleled those of H9N2 virus (10) and therefore indicated the hotspots for viral evolution and surveillance in China. Further, we presented the H7N9 propagation network based on the phylogeny of all the internal genes, which provided a more comprehensive diffusion pattern compared with the former graph derived from only NA gene segment (4). This may reflect the fact that the internal gene segments affected by both reassorment and poultry movements displayed more diversity than the surface genes.

Given the higher substitution rate of both H7N9 and H9N2 isolates during 2013–2014, LPMs had broad potential to affect viral mergence and evolution. Specifically, the co-circulation and co-infection of different subtypes can provide some cross-immunity to infections from other subtypes, and lower the infection rate, the number of susceptibles, and the consequent probability of pandemic emergence. However, it may also result in the viral evasion from host immunity response through continuous and rapid mutations to better adapt to and efficiently transmit in new environment. Still, the highly-substituted internal genes of H7N9 viruses indicated that this novel AIVs was in the expansion period during 2013–2014. This inference was rational and supported by the modeling work where viral lineage from epidemic regions was found to have higher substitution rate than those from endemic regions (36). Therefore, compared with the inter-regional viral dynamics facilitated by LPTs, LPMs served as the local reservoir for the emergence and evolution of novel viruses.

In the perspective of live poultry trading network in the spatially-structured spread of AIVs presented here, additional interventions should be jointly implemented to restrict viral expansion along LPTs paths and targeted at live poultry workers. Despite the effectiveness in reducing the daily number and growth rate of new human cases (21, 22) and the amount and detection rate of viable viruses (37) the mandatory closure of LBMs alone was unlikely to eliminate the zoonotic threat (38) accounting for the LPTs as the pathway with the highest likelihood of viral spread^2^. It is also reported that humans who engaged in the transportation work of live chickens and ducks was particularly susceptible to infections from AIVs (39). These facts ascertained the role of live poultry trading in the spillover to humans at avian–human interface and the occurrence of H7N9 human cases. Therefore, a multi-sector, cost-effective approach and even international collaboration will be essential for the substantial reduction in the risk of disease spread and the build of a safer trade in animals^3^.

These findings were based on the assumptions themed around our research interests which should be taken into account when making interpretations and generalizations. Firstly, since we focused on the inter-provincial or macroscopic live poultry trading patterns and genetic differentiation of H7N9 viruses, we assume that the inter-provincial/regional LPTs occurred only along the national roads connecting pairwise locations. Similarly, market accessibility was also defined by the density of national road. However, this dominant role of national road was scale-dependent and cannot be directly generated to other spatial scales or the contribution of other factors. More precisely, previous studies have shown that detailed and localized LPTs along provincial roads (40), illegal trades (38), and even cross-border transports^4^ have also made crucial contributions to shaping the viral diffusions. Additionally, although our inferences were implemented using the integrated molecular dataset in Bayesian statistical framework, sampling bias may still have potential effect on the reconstructed phylogeny and the inferred transmission networks. Whereas, the inference system was partially evaluated, our study demonstrated the role of the live poultry trading in dominating the spatially-structured evolution and expansion of H7N9 viruses and the need for active surveillance and interventions on poultry trading.

## Material and data availability

The genetic sequence data analyzed for this study can be found in the GenBank database of the National Center for Biotechnology Information (http://www.ncbi.nlm.nih.gov/pubmed/). The poultry production and consumption data are available from the China Statistical Yearbook (http://www.stats.gov.cn/english/Statisticaldata/AnnualData/).

The H9N2 sequence data collected in Jiangxi Province, poultry transportation and the national road data supporting the conclusions of this manuscript are available on request.

## Author contributions

RL and BX deigned the study. TZ and JC performed the field and laboratory experiments. HL and YW provided the geographic information data. RL, TZ, YBai, YBi, JC, and BX analyzed and interpreted the data. RL and BX wrote the paper.

### Conflict of interest statement

The authors declare that the research was conducted in the absence of any commercial or financial relationships that could be construed as a potential conflict of interest.

## Footnotes

^1^FAO. H7N9 situation updates. (2016) Available online at: http://www.fao.org/ag/againfo/programmes/en/empres/H7N9/situation_update.html

^2^FAO. Qualitative risk assessment update. Addressing avian influenza A(H7N9). Emergency Prevention System (EMPRES) Program. Rome. (2014) Available online at: http://www.fao.org/3/a-i3813e.pdf

^3^FAO. Viet Nam – China aim for safer trade of animals and animal products. (2016) Available online at: http://www.fao.org/vietnam/news/detail-events/en/c/381699/

^4^FAO. Approaches to controlling, preventing and eliminating H5N1 highly pathogenic avian influenza in endemic countries. (2011) Available from: http://www.fao.org/docrep/014/i2150e/i2150e.pdf
