# Supplementary material for: Live Poultry Trading Drives China's H7N9 Viral Evolution and Geographical Network Propagation
Source: Front Public Health. 2018 Jul 27;6:210. doi: 10.3389/fpubh.2018.00210 (PMC6094976; doi:10.3389/fpubh.2018.00210)
Supplement: Supplementary file 1 [file Data_Sheet_1.pdf]

frontiers

Supplementary Material

Live Poultry Trading Drives China's H7N9 Viral Evolution and Geographical Network Propagation

Ruiyun Li[1,†], Tao Zhang[2], Yuqi Bai[2], Haochuan Li[3], Yong Wang[4], Yuhai Bi[5], Jianyu Chang[6*], Bing Xu[1,2*]

*** Correspondence:** Bing Xu: bingxu@tsinghua.edu.cn; Jianyu Chang: changjianyu@cau.edu.cn

1 Supplementary Figures and Tables

1.1 Supplementary Figures

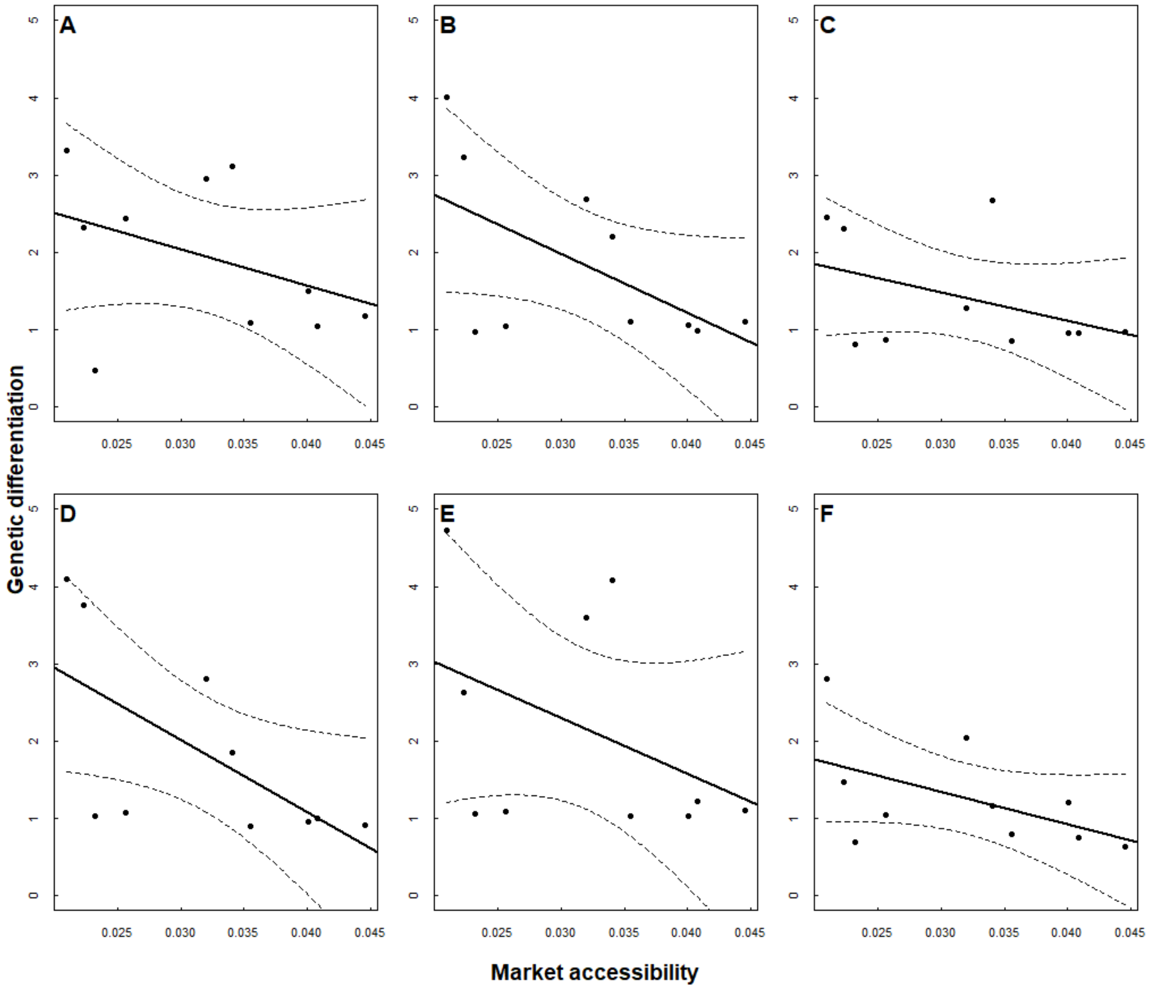

Supplementary Figure 1. Relationship between the level of genetic differentiation and market accessibility. For each internal gene segment—(A) PB2, (B) PB1, (C) PA, (D) NS, (E) NP and (F)

MP, the genetic differentiation level of the internal gene segments of H7N9 isolates was measured by F_{ST} index and then transformed into $F_{ST}/(1-F_{ST})$ when plotted against the market accessibility. Linear regression and 95% confidence interval were indicated by solid and dot lines, respectively.

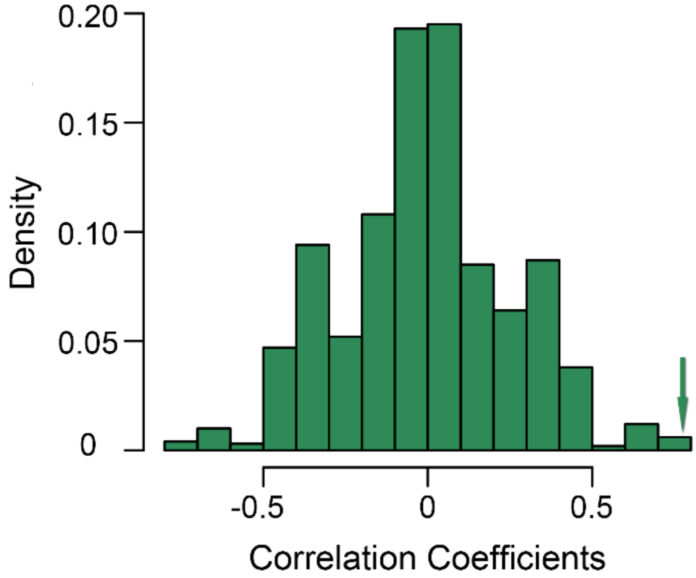

Supplementary Figure 2. Distribution of correlation coefficients for the randomly-generated networks. The histogram of correlation coefficients was constructed based on the 1,000 random networks, and the coefficients of viral migration and live poultry transportation network was indicated by the arrow.

1.2 Supplementary Tables

Supplementary Table 1. Lineage distribution of H9N2 internal gene segments. The similarity between H7N9 virus and H9N2 lineages was quantified by the average genetic distance. The H9N2 lineage shared the highest similarity with H7N9 virus was defined as the closest lineage. The presence of each H9N2 lineage were indicated by “+”; whereas those closest H9N2 lineages were highlighted by “++”.

Lineage	Representative virus	PB2	PB1	PA	NS	NP	MP
HK/G1/97	A/quail/Hongkong/G1/97	+	+	+	+	+	++
BJ/1/94	A/chicken/Beijing/1/94	+	+	+	++	+	+
HK/289/78	A/duck/Hongkong/289/78	+	+	+	+	+	+
HK/AF157/92	A/quail/Hongkong/AF157/92		+		+	+	+
SH/F/98	A/chicken/Shanghai/F/98	+	++	++		++	
ST/163/04	A/duck/Shantou/163/2004	++					
ST/5663/01	A/quail/Shantou/5663/2001			+			
HK/Y439/97	A/duck/Hongkong/Y439/97			+	+	+	
KR/96323/96	A/chicken/Korea/38349-p96323/96	+	+	+	+	+	+
Hok/49/98	A/duck/Hokkaido/49/98		+	+			
IL/90658/00	A/chicken/Israel/90658/2000		+		+		
DE/113/95	A/Duck/Germany/113/95	+	+	+		+	+
PK/UDL-01/05	A/chicken/Pakistan/UDL-01/2005	+					
CA/189/66	A/turkey/California/189/66				+		
WI/1/66	A/turkey/Wisconsin/1/1966	+	+	+	+	+	+

Supplementary Table 2. Statistical performance of molecular evolutionary models. Model comparison and selection was based on Bayes factor with different combination of molecular clock model (i.e., strict and relaxed clock model) and coalescent prior (i.e., Bayesian Skyline Plot (BSP), constant size and exponential growth).

Molecular clock model	Coalescent prior	Estimated marginal likelihood	Bayes factors					
			Strict			Relaxed		
			BSP	Constant	Exponential	BSP	Constant	Exponential
Strict	BSP	-6018.39	-	-13.26	9.03	-108.56	-98.91	-99.67
	Constant	-6005.13	13.26	-	22.28	-95.30	-85.65	-86.41
	Exponential	-6027.42	-9.03	-22.28	-	-117.58	-107.94	-108.69
Relaxed	BSP	-5909.84	108.56	95.30	117.58	-	9.65	8.89
	Constant	-5919.48	98.91	85.65	107.94	-9.65	-	-0.76
	Exponential	-5918.73	99.67	86.41	108.69	-8.89	0.76	-

Supplementary Table 3. The number of live poultry markets in each province searched with different keywords.

Keywords	Shanghai	Jiangsu	Zhejiang	Guangdong	Jiangxi
Live poultry	1	1	1	0	1
Agricultural market	329	2236	1608	898	347
Fresh market	5	7	0	4	8
Live bird market	0	5	1	8	2
total	335	2249	1610	910	358
